# SUBJECTIVE COGNITIVE DECLINE IN CAREGIVERS: 21 STATES, PUERTO RICO, AND DC, 2015-2017

**DOI:** 10.1093/geroni/igz038.675

**Published:** 2019-11-08

**Authors:** Christopher A Taylor, Valerie J Edwards, Kenneth A Knapp, Erin D Bouldin, Lisa C McGuire

**Affiliations:** 1 Centers for Disease Control and Prevention, Atlanta, Georgia, United States; 2 Alzheimer's Disease and Healthy Aging Program, Centers for Disease Control and Prevention, Atlantta, Georgia, United States; 3 New York Medical College, Valhalla, New York, United States; 4 Appalachian State Unversity, Boone, North Carolina, United States; 5 Alzheimer's Disease and Healthy Aging Program, Centers for Disease Control & Prevention, Atlanta, Georgia, United States

## Abstract

Informal caregivers can provide assistance that can help family members and friends live in the community longer but can place caregivers at increased risk for poorer health outcomes. Subjective cognitive decline (SCD) is the self-reported experience of worsening or more frequent confusion or memory loss. The objective of this study is to describe SCD in caregivers. Data were analyzed from 21 states, Puerto Rico, and District of Columbia who administered both the Caregiver and Cognitive Decline modules of the Behavioral Risk Factor Surveillance System in the same year for 2015–2017. A higher proportion of caregivers reported SCD (13.4%) compared to non-caregivers (10.2%). Of those who did need assistance with daily activities due to SCD, 1 in 8 non-caregivers were unable to the necessary assistance compared to 1 in 4 caregivers. SCD among caregivers is of particular concern because it affects both the caregiver and care recipient.

